# Proteomic analysis of ‘Zaosu’ pear (*Pyrus bretschneideri* Rehd.) and its red skin bud mutation

**DOI:** 10.1186/1477-5956-10-51

**Published:** 2012-08-29

**Authors:** Min Hu, Zonghao Qiu, Peng Zhou, Lingfei Xu, Junke Zhang

**Affiliations:** 1 College of Horticulture, Northwest A & F University, Yangling, Shaanxi, 712100, China; 2 Key Laboratory of Horticultural Plant Biology and Germplasm Innovation in Northwest China, Ministry of Agriculture, Yangling, Shaanxi, 712100, China

**Keywords:** Pear, Red skin bud mutation, Plant proteomics, MALDI TOF/TOF-MS

## Abstract

**Background:**

Breeding for strong red skin color is an important objective of the pear breeding program. There are few reports of proteome research in green skin pear and its red skin bud mutation. The manuscript at hand is one of the first studies dealing with 2D-PAGE-based analysis of pear fruits and leaves, establishing a suitable sample preparation and testing different 2D-PAGE protocols. Therefore, it may grant a basis for further studies on the pear proteome being the studies main goal. A proteomic analysis was conducted on leaves and fruits of ‘Zaosu’ pear (*Pyrus bretschneideri* Rehd.) and its red skin bud mutation in order to reveal their genetic differences in the protein level.

**Results:**

In the present study, the optimized two-dimensional (2-D) gel electrophoresis system of pear leaf and fruit was set up, and applied to analyze the leaves and fruit protein. The interesting peptide fragments were determined using 4800 Plus MALDI TOF/TOFTM Analyzer mass spectrometer, and the sequence obtained was blasted in NCBInr to identify the differentially-expressed protein. In the 1.5-fold differently-expressed proteins between ‘Zaosu’ pear and its mutant, 10 out of 35 proteins in fruit and 12 out of 24 ones in leaves were identified successfully. Among the 22 identified proteins, 7 protein spots were related to photosynthesis and energy metabolism; 4 were associated with environmental stress; 4 with disease defense; 2 with amino acid metabolism; 2 with cytoskeleton; 1 with antioxidant function; 1 with calcium metabolism; and 1 with unknown function. Moreover, related physiological index, such as chlorophyll content, Rubisco content and polyphone oxidase activity, were different between ‘Zaosu’ pear and its mutant.

**Conclusion:**

A 2-D gel electrophoresis system of pear leaves and fruits was established, which was suitable for the analysis of proteome comparison. To the best of our knowledge, we have performed the first analysis of the proteomic changes in leaves and fruits of ‘Zaosu’ pear and its red skin bud mutation. Our study provides important information on the use of proteomic methods for studying protein regulation of ‘Zaosu’ pear and its red skin bud mutation.

## Background

**Figure 1:**
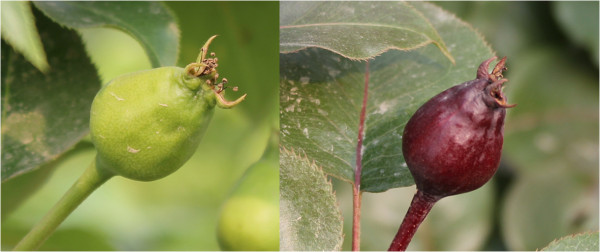
Phenotypes of ‘Zaosu’ pear and its red bud mutation.

Pear is one of the most important fruit crops in the world. Breeding for strong red skin color is an important objective of the HortResearch interspecific pear breeding program [1]. The Asian pear cultivar ‘Zaosu’ (*Pyrus bretschneideri* Rehd.) was commercialized in China. A bud mutation of ‘Zaosu’ pear which caused the red skin pigmentation was discovered in Shaanxi Province, China (Figure 1) [2]. The ‘Zaosu’ pear fruit is green at maturity, and then turns yellow when fully ripe. In contrast, this bud mutation fruit is red throughout the whole maturation stage, that is, its young leaves, flowers, buds and fruits are red. Through observation and field experiment for many years, the red skin character of this bud mutation has been stable. At present, there are some red pear germplasm resources in China, but such kind of bright color, high quality and large fruit-shaped cultivars are very scarce, and the European red pear cultivars are soft flesh texture. Therefore, this mutation is considered to be a unique and valuable germplasm resource of pear.

Since the proteomic approaches have been applied in fruit tree science, more and more researchers began to pay attention and actively participate in the proteomics. Prinsi et al. [3] performed a proteomic analysis on peach fruit mesocarp, and they set up a suitable protocol for improving protein extraction from peach mesocarp, and identified 53 differently abundant spots by LC-ESI-MS/MS. Muccilli et al. [4] used 2-DE with LC-MSMS to identify the differentially expressed proteome of a pigmented sweet orange (*Citrus sinensis*, cv. Moro) in comparison with a common cultivar (Cadenera), and identified 55 differentially expressed proteins. Feng et al. [5] studied the differential expression of protein in red pear after the bagging treatment, and they found 35 protein spots, and 21 spots were identified and classified into functional classes. Negri et al. [6] reported that analysis of the different proteins in the grape ripening process revealed 80 different-expressed protein spots, applied a two-way hierarchical clustering analysis to the proteins, and identified 69 proteins by LC-ESI-MS/MS. Martinez-Esteso et al. [7] investigated the changes in the extracellular proteome of a grapevine (*Vitis vinifera* cv. Gamay) cell suspension in response to elicitation with methylated cyclodextrins (MBCD) and methyl jasmonate, and identified 25 proteins by MALDI-TOF. These studies provided valuable experience for the subsequent researchers. At present, however, no studies have been reported to date of the differential expression of protein in green skin pear and its red skin bud mutation.

In the present study, we used young leaves and fruits of ‘Zaosu’ pear and its red skin bud mutation as materials to develop an efficient two-dimensional (2-D) gel electrophoresis system, and find the differently-expressed proteins with mass spectrometer. The results may reveal their genetic differences in the protein level.

## Results and discussion

### The set screening of 2-DE gel electrophoresis for leaves and fruits of pear

**Figure 2:**
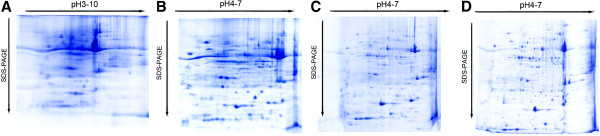
**Two-dimensional gel images using different pH IPG and IEF procedures. A**. 7 cm pH 3-10; **B**. 7 cm pH 4-7; **C**. 17 cm IEF Procedure 1; **D**. 17 cm Procedure 2.

In order to select the most suitable parameters for 2-DE gel electrophoresis for leaves and fruits of pear, we used IPG strips with 7 cm pH 4-7 and pH 3-10 combined with IEF Procedure 2. The proteomic analysis with pH 3-10 showed that the pear protein spots distributed mainly within the range of pH 4-7 (Figure 2A). Importantly, 282 protein spots were detected on pH 3-10 IPG strip, while 377 spots were on pH 4-7 IPG strip (Figure 2B). For total cellular protein samples from pear leaves, pH 4-7 IPG strip was applied and 500 μg of protein sample was loaded and rehydrated, then IEF Procedure 1 or 2 was applied to obtain the following 2-D gel images (Figure 2C, D). The detected protein spots were 163 and 252 using Procedure 1 and 2, respectively. Therefore, IEF Procedure 1 with relatively high quality of proteomic image was selected for the further study.

### Comparative analysis of 2-DE gel from leaves and fruits of ‘Zaosu’ pear and its red skin bud mutation

**Figure 3:**
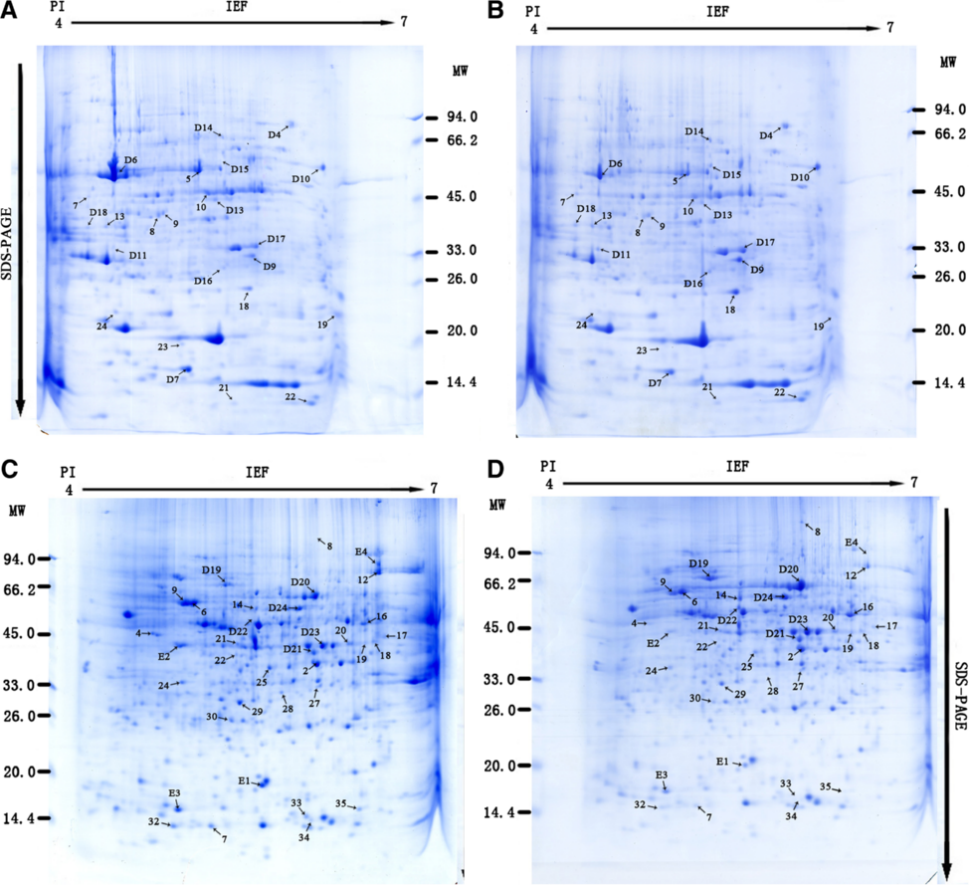
**Two-dimensional images of ‘Zaosu’ pear and its red mutation. A**. leaf of ‘Zaosu’ pear; **B**. leaf of the red mutation **C**. fruit of ‘Zaosu’ pear; **D**. fruit of the red mutation.

**Figure 4:**
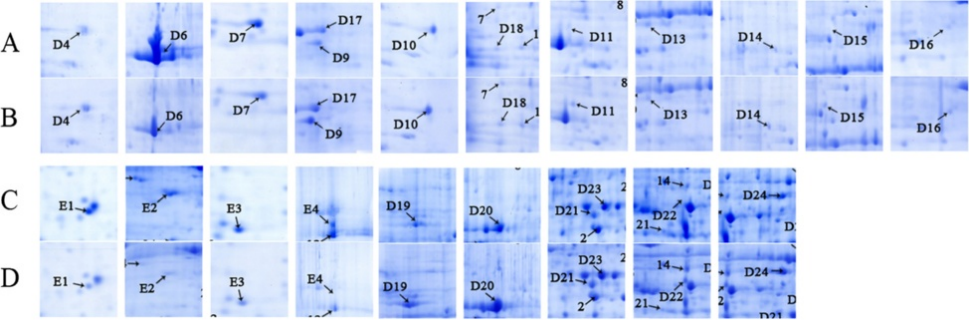
**Magnified Images of the differential proteins between ‘Zaosu’ pear and its red mutation.** The 22 successful identified protein spots in ‘Zaosu’ pear and its red mutation. **A**: leaf of ‘Zaosu’ pear; **B**: leaf of the red mutation; **C**: fruit of ‘Zaosu’ pear; **D**: fruit of the red mutation.

**Figure 5:**
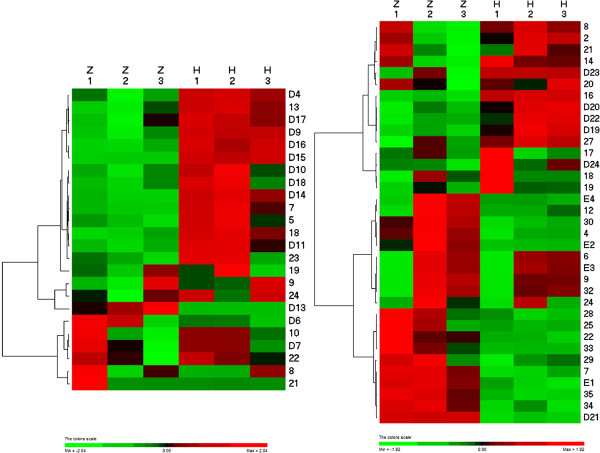
**Clustering analysis of 2-DE gel data.** Data from 24 spots and 35 spots (the spot IDs such as D* and E* has been detected by MALDI-TOF-TOF/MS) that showed an at least 1.5-fold change in their relative volume between ‘Zaosu’ pear and its red skin bud mutation leaves and fruits (A comes from leaves and B comes from fruits), were subjected to two-way hierarchical cluster analysis, performed with PermutMatrix. Pearson’s distance and Ward’s algorithm were used. Each coloured cell represents the average of the relative spot volumes, according to the colour scale depicted at the bottom of the figure.

We conducted the analysis on 2-DE gels between ‘Zaosu’ pear leaves (Figure 3A), fruits (Figure 3C) and the red skin bud mutation leaves (Figure 3B), fruits (Figure 3D) by PDQuest 2-DE 8.0.1 software. There are 24 protein spots detected whose normalized volumes were in at least a 2:1 ratio between the two leaves samples (Among them, 15 spots were up-regulated in the red skin bud mutation, 6 spots were down-regulated. There is 1 spot specifically expressed in the red skin bud mutation, 2 spots specifically in ‘Zaosu’ pear.), and 35 protein spots detected between the two fruits samples (Among them, 15 spots were up-regulated in the red skin bud mutation. 12 spots were down-regulated. There is 6 spots specifically expressed in the red skin bud mutation, 2 spots specifically in ‘Zaosu’ pear). After we conducted the cluster analysis of the peak value which showed in the results of PDquest software, 15 spots from leaves and 10 spots from fruits were chosen for MALDI-TOF-TOF/MS analysis. The results indicated that 12 spots in leaves were successfully identified, among the 12 spots, 8 spots (D4, D9, D10, D11, D14, D16, D17 and D18, Figure 4) were up-regulated, 3 spots (D6, D7 and D13, Figure 4) were down-regulated and 1 spot (D15, Figure 4) specifically expressed in the redskin bud mutation. 10 spots in fruits were successfully identified, among 10 spots, 3 spots (E1, E2 and E3) down-regulated, 5 spots (D19, D20, D21, D23, D24, Figure 4) up-regulated, 1 spots (E4) only exist in ‘Zaosu’ and 1 spot (D22, Figure 4) only exist in the red skin bud mutation. Cluster analysis of 2-DE gel data was performed with PermuMatrix (Figure 5), and in generally the figure can reflect the protein expression more intuitively.

### MALDI-TOF-TOF-MS/MS Analysis and protein identification

Generally, 22 spots were successfully identified in leaves and fruits of ‘Zaosu’ pear and its red skin bud mutation. The basic information of identified protein was listed in Table 1, such as pI, retrieval registration number, protein score, protein molecular weight, and protein name. D4-D18 are information from leaf proteins, while D19-E4 are information from fruit protein. D16 and E3 are EST sequences. In order to conduct further study about the changes of physiological and biochemical parameters by the red skin buds mutation, we get a more detailed protein information by searching for relevant literature. Heat shock protein 70 (D4) was identified from cucumber. It has 23 peptides matched, and the protein was up-regulated in the red skin bud mutation. HSP70 is a family of ubiquitously expressed heat shock proteins. The HSP70s are an important group of family members of the cell’s machinery for protein folding, and help to protect cells from stress. Rrubulose-1, 5-bisphosphate carboxylate/oxygenize large subunit (D6) was identified from *hydrangea arborescens*. It has 21 peptides matched, and the protein was down-regulated in the mutant. It is most commonly known by the shorter name RuBisCo, which is an enzyme involved in the Calvin cycle that catalyzes the first major step of carbon fixation. Ribulose-1, 5-bisphosphate carboxylate/oxygenize large subunit precursor (D7) was identified as from *Liquidambar styraciflua*, with 14 matched peptides. It was down-regulated in the mutant. After being modified by related enzymes, it becomes RuBisCo. LHC-type-chlorophyll-a/b binding protein (D9) was identified from mung bean, with 3 matched peptides. It was up-regulated in the mutant. The overall structure of PSII is known to be extremely complex with six different pigment-binding subunits having purely antenna function and an additional subunit binding the RC pigments. The outer or peripheral antenna, which consists of a family of chl a-b-binding proteins, is known to be responsible for about 60% of total light absorption in PSII. Calreticulin (D10) was identified from *Prunus serrulata*, with 14 matched peptides. It was up-regulated in the mutant. Calreticulin is an essential Ca^2+^-binding/storage chaperon resident protein of endoplasmic reticulum or sarcoplasmic reticulum existing in a diverse range of species. The protein is involved in the regulation of intracellular Ca^2+^ homeostasis and endoplasmic reticulum Ca^2+^ storage capacity, and is also an important molecular chaperone involved in “quality control” within secretory pathways. Putative pyridoxine biosynthesis protein isoform A (D11) was identified from tobacco, with 8 matched peptides. It was up-regulated in the mutant. This protein was mainly related to plasma membrane and end membrane system, as a rate-limiting enzyme in the synthesis of vitamin B6. Ribulose diphosphate carboxylate/oxygenize activates, chloroplast (D13) was identified from *Buschohne*, with 7 matched peptides. It was down-regulated in the mutant. RubisCO activates is considered to function as catalysis of Rubisco for Carbinol and binding to Mg^2+^ to dynamic. Mitochondrial heat shock 70 kDa protein (D14) was identified from *Buschohne*, with 12 matched peptides. It was up-regulated in the mutant. The HSP70 located in the mitochondria not only has the molecular chaperone function [8], but also improves the resilience of plant and protect the electron transport during the process of oxidative phosphorylation [9,10]. Os03g0718100 (D15) was identified from Japanese rice, it has 14 peptides were matched. Only exist in the red skin bud mutation. It is one kind of actin which was regarded as an internal standard, and class belongs to the housekeeper protein, acts as a load controllers. Pbzs315 (D16) was identified as EST sequence from ‘Zaosu’ pear, with 6 matched peptides. It was up-regulated in the mutant. Its biological function was to improve the disease resistance. Oxygen-evolving complex protein 1 (D17) was identified form rice, with 11 matched peptides. It was up-regulated in the mutant. Its main function involves in the process of photosynthetic oxygen evolution, as manganese containing external membrane protein. NAD-dependent maleate dehydrogenase (D18) was identified form peach, with 8 matched peptides. It was up-regulated in the mutant. Maleate dehydrogenase is an enzyme in the citric acid cycle that catalyzes the conversion of maleate into oxaloacetate (using NAD^+^) and *vice versa*, mainly involved in energy metabolism. A high molecular weight heat shock protein (D19) was identified from Apple, with 24 matched peptides. It was up-regulated in the mutant. Generally, heat shock protein which greater than HSP70 we called high molecular weight heat shock protein. It is associated with stress resistance. Polyphone oxidase precursor (D20) was identified from apricot, with 4 matched peptides. It was up-regulated in the mutant. It is the precursor of polyphone oxidase, which catalyzes the o-hydroxylation of monophenols to o-diaphanous. It may also be referred as tyrosinases. (D21) was identified as coffee acid 3-O-methyltransferase, from apricot, with 7 matched peptides. It was up-regulated in the mutant. It controls S-lignin (lignin monomer) specific pathway, as well as improves disease resistance. Actin (D22) was identified from Gossypium hirsutum, with 16 matched peptides. It was only exist in the red skin bud mutation. It is an important component of plant cytoskeleton, and has important significance in maintaining the normal physiological and biochemical processes. S-adenosylmethionine synthase 2 (D23) was identified from *Autumn Oleaster*, with 14 matched peptides. It was up-regulation in the mutant. It is an enzyme which catalyses the synthesis of S-adenosylmethionine (SAM) from methionine and ATP. Polyphone oxidase 2 precursor (D24) was identified from apple, with 7 matched peptides. It was up-regulated in the mutant. It is one of precursor of polyphone oxidase. Ribulose 1,5-bisphosphate carboxylate (E1) was identified from *Loeseneriella* A.C. Smith, with 10 matched peptides. It was down-regulation in the mutant. It is an important regulatory enzyme of photosynthetic carbon metabolism. Heat shock cognate 70 kDa protein 2 (E2) was identified from tomato, with 22 matched peptides. It is specially expressed in ‘Zaosu’ pear. It is one of HSP70 family, and can be markedly induced under stress. Ipa. ADAB-aaa60g07.b1 (E3) was identified as EST sequence from peanut, with 11 matched peptides. It was down-regulation in the mutant. It is an expressed sequence tag. Putative methionine synthase (E4) was identified from Arabidopsis, with 12 matched peptides. It is specifically expressed in ‘Zaosu’ pear. Methionine synthase is responsible for the regeneration of methionine from homocysteine. It also participates in the S-adenosylmethionine biosynthesis and regeneration cycle.

**Table 1:** Proteins result analyzed and identified by MALDI-TOF-TOF/MS

***Spot no***	***Protein name***	***Accession no.***	***Protein MW/PI***	***Pep. count***	***Proten score***	***Ratio ‘Zaosu’/the red mutant***	***Relative protein content***
*Photosynthetic and energy metabolism proteins*							
D6	ribìlose-1,5-bisphosphate carboxylase/ oxygenase large subunit	gi|15148414	52493.4/6.32	21	702	2.57	
D7	ribìlose-1,5-bisphosphate carboxylase/ oxygenase large subunit Precursor	gi|7008057	53067.6/6.04	14	520	1.62	
D9	LHCII type I chlorophyll a/b binding protein	gi|8954293	27900.1/5.29	3	102	0.47	
D13	Ribìlose bisphosphate carboxylase/ oxygenase activase	gi|10720248	48341.7/8.19	7	322	5.97	
D17	oxygen-evolving complex protein 1	gi|739292	26603.5/5.13	11	576	0.42	
D18	NAD-dependent malate dehydrogenase	gi|15982948	35817.5/6.6	8	255	0.49	
E1	ribìlose1,5-bisphosphate carboxylase	gi|9909955	52296.2/6.04	10	305	2.60	
*Stress-related proteins*							
D4	Heat shock protein 70 [Cucumis sativus]	gi|1143427	75480/5.15	23	850	0.51	
D14	shock 70 kDa protein, mitochondrial	gi|399940	72720.6/5.95	12	98	0.37	
D19	high molecìlar weight heat shock protein	gi|6969976	71570.4/5.17	24	743	0.37	
E2	Heat shock cognate 70 kDa protein 2	gi|123620	71062/5.08	22	555	3.47	
*Disease resistance-related proteins*							
D16	Pbzs315 SSH cDNA library of pear cv. ‘Zaosu’ inocìlated with Venturia nashicola Pyrus x bretschneideri cDNA, mRNA sequence	gi|255987829	35747.7/9.88	6	279	0.33	
D20	polyphenol oxidase precursor	gi|3282505	67432.9/6.39	4	273	0.47	
D21	S-adenosysl-L-methionine:caffeic acid3-O-methyltransferase	gi|3913295	40135.3/5.52	7	247	0.38	
D24	polyphenol oxidase 2 precursor	gi|14194273	65762/6.09	7	178	1.72	
*Cytoskeleton-related proteins*							
D15	Os03g0718100	gi|115454971	42014/5.3	14	255	Only observed in the red mutant	
D22	actin	gi|32186902	42057.1/5.3	16	531	Only observed in the red mutant	
*Amino acid metabolism-related proteins*							
D23	S-adenosylmethionine synthase	gi|75308025	43564.9/5.5	14	635	0.53	
E4	putative methionine synthase	gi|14532772	84899.8/6.09	12	148	Only observed in ‘Zaosu’ pear	
*Antioxidant-related proteins*							
D11	Putative pyridoxine biosynthesis protein isoform A	gi|46399269	33353.1/5.93	8	218	0.24	
*Calcium-related proteins*							
D10	Calreticìlin	gi|11131904	48557.5/4.4	14	380	0.49	
*Others*							
E3	Ipa. ADAB-aaa60g07.b1 IPA1-seed Arachis ipaensis cDNA 5', mRNA sequence	gi|296593933	23794.4/9.29	11	92	1.60	

After being identified by mass spectrometry and queried the protein database, all identified proteins were classified into the following groups according to biological function: photosynthetic and energy metabolism proteins, antioxidant proteins, anti-stress proteins, amino acid metabolism proteins, cytoskeleton related proteins and Calcium-related proteins.

### Photosynthetic and energy metabolism proteins

There are 7 proteins (D18, D6, D7, E1, D13, D9 and D17) which take part in photosynthetic and energy metabolism. Among the identified proteins, D6, D7, D13 and E1 were all down-regulated in red mutant, D9, D17 and D18 were up-regulated. Light-harvesting pigment system consists of a variety of colors and protein complexes. Normal (green) leaves contain more chlorophyII, while yellow leaves contain more caroteoids. When there are more soluble sugars *in vivo*, more anthocyanin would be formed and leaves become red. The light absorbed by anthocyanin is not used for photosynthesis. Photo system II (PSII) includes the reaction center, light-harvesting complex II (LHC II) and a manganese cluster of oxygen evolving complex. The main function of oxygen evolving complex in light-harvesting pigment system is to harvest light, and to transfer light to the reaction center pigment. Chlorophyll a/b binding protein performs the function of pigment molecules. LHC II in chloroplast thylakoids is the most abundant protein as an antenna, and it has four functions as follows: to transfer and harvest light, distribute and balance the energy between PSII and PSI, protect light and excess energy dissipation, and maintain the structure of thylakoid [11-15]. Oxygen-evolving complex protein makes thermodynamically-stable water lionize and release oxygen in relatively mild conditions, and a catalyst must be involved this reaction [16-19].

Ribulose-1,5-bisphosphate carboxylate/oxygenize is a bifunctional enzyme [20], which catalyzes reaction of RuBP carboxylation in C3 pathway. It is the key enzyme in photosynthetic carbon assimilation, and catalyzes the RuBP oxygenize reaction in photorespiration. Therefore, the enzyme in the regulation of photosynthesis and photorespiration rate is crucial to the net photosynthethesis. RuBisCO requires a phthalocyanine of ammonia and Mg^2+^ binding for the activity of catalytic. RuBisCO activates was the chloroplast enzyme coded by nuclear genes [21]. It can remove sugar phosphate inhibitors on RuBisCO active site, and catalyzes CO_2_ and Mg^2+^ to bind with RuBisCO. The activates is now identified to be a member of the AAA^+^ family, whose members participate in macromolecular complexes that perform diverse chaperon-like functions [22].

NAD-dependent maleate dehydrogenase (MDH) is an enzyme commonly existing in animals, plants and microorganisms. MDH mainly involves in the tricarboxylic acid cycle, reactive oxygen species (ROS) metabolism and energy metabolism of mitochondria in plant. It is also a key enzyme for oxaloacetate regeneration, and catalyzes H^+^ of hydroxyl in malice acid detected to NAD(P)^+^ and generates oxaloacetate, which is a reversible reaction. In this study, NAD-dependent maleate dehydrogenase was upregulated in the mutant, suggesting that respiratory metabolism of the red skin bud mutation might be enhanced.

In the present study, proteins related to light reaction were identified: oxygen-evolving complex protein 1 (D17) and LHCII type I chlorophyll a/b binding protein (D9). Both of them were up-regulated in the red skin bud mutation. As the two proteins were important in Photo System II (PSII), we assume that PSII increased in the bud mutation. PSII is the most important light reaction center and LHC II is the most abundant light-harvesting complex in chloroplast thylakoids, the higher expression of these proteins may contribute to enhancement of the ability to the light reaction stage. Interestingly, 4 photosynthesis Calvin cycle related protein (D6, D7, D13, E1), Rubisco (in leaf), Rubisco (in fruit), Rubisco Precursor and Rubisco activates, were down-regulated in the mutant. Rubisco is the key regulatory enzyme in the ‘light-independent reactions’ of the photosynthesis Calvin cycle [23]. Both of Rubisco precursor and Rubisco activates were down-regulated, suggesting the decline of carbon reaction. We speculate that there are a large number of anthocyanins in the red skin bud mutation, and the light absorption by anthocyanin is not used for photosynthesis, and only stays in light reaction stage, but does not take part in carbon reaction, resulting in the improvement of the ability of light reaction and decrease of the ability of carbon reaction. Moreover, it was found that PPO precursor expression was up-regulated in the mutant, as well as the enzyme activity, indicating that the red bud mutation may has stronger disease resistance. However, the detailed mechanism is not clear.

### Stress-related proteins

There were 4 stress-related proteins detected as followed: D4, heat shock protein 70 (HSP70); D14, heat shock protein 70 in mitochondrial; D19, high molecular weight heat shock protein; E2, heat shock cognate 70 kDa protein 2; D19 and E2 originate from fruit, and D4 and D14 originate from leaf. Among them, D4, D14 and D19 were all up-regulate and E2 was down-regulate. All of them belong to the same shock protein family. Heat shock protein is one kind of conservative family protein. Stress protein will be synthesized when organism faces stress factors such as heavy metal, hypoxia, high temperature and frost [24,25]. HSP often have a molecular chaperone function [8]. However, heat shock proteins in mitochondrial (MT-HSP) also have the function that other small molecules do not have, such as improving the heat resistance and the cold resistance of plants [10]. In addition, in the process of oxidative phosphorylation MT-HSP protects the electron transport [26]. Researchers have found that HSP90 not only exist in the cytoplasm, but also in the nucleus and endoplasmic reticulum [27]. HSP70 is able to participate in disposal of damaged or defective proteins. Interaction with CHIP (Carboxyl-terminus of HSP70 interacting protein)-an E2 ubiquitin ligase allows HSP70 to pass proteins to the cell’s ubiquitination and proteolysis pathway [26,28]. In this study, 4 protein members in heat shock protein family were detected. They are in cytoplasmic and organelle from both fruits and leaves. E2 is unique in ‘Zaosu’pear and the other three are up-regulated in the red skin bud mutation. As expected, the red skin bud mutation has greatly improved resistance ability and acts an active role in regulation of cell death and error protein degradation.

### Disease resistance-related proteins

4 proteins related to disease resistance were detected as followed: D16, pbzs315; D20, polyphone oxidase precursor; D21, coffee acid 3-O-methyltransferase (COMT); D24, polyphone oxidase 2 precursor; D16 originates from leaves, and D20, D21 and D24 originate from fruit. All of them were identified up-regulate in “Zaosu” red bud mutant. D16 is a EST sequence. Polyphone oxidase (D20) is combined with thylakoid membranes in plant tissue. It is a tetramer which contains four atoms of copper per molecule, and binding sites for two aromatic com pounds and oxygen. The enzyme catalyzes the o-hydroxylation of monophenols to o-diaphanous. The substances containing o-diaphanous have the function of disease resistance. Pathogen-related protein PPO protects cells against the pathogens by catalyzing synthesis of lignin and quinones. The PPO activity also showed higher in the mutant. Moreover, there are 2 spots detected to be up-regulated in the fruit of redskin bud mutation, which indicates the enhancement in disease resistance of redskin bud mutation. COMT (D21) is a key enzyme in lignin-specific synthesis pathway to regulate the specific synthesis of syringe lignin monomers. The enzyme catalyzes caffeicacid to ferulic acid, and 5-hydroxyl ferulic acid to sinapic acid. In the process of plant growth and development, lignin gradually penetrated into the cell wall, and increased the hardness of the cell wall. Lin YZ [29] reported that COMT participated in the biosynthesis and transportation of lignin in the probenazole-induced resistance in rice. In our study, it was found that COMT was up-regulated in the mutant, indicating that ‘Zaosu’ Red may have stronger lignin synthesis ability. PBZS315 is a cDNA squequence, which is a disease-resistance gene find in the study of pear scab (*Venturia pyrina* Aderh.). This gene was up-regulated in the red skin bud mutation, which may contribute to the enhancement of red skin bud mutation’s disease resistance. Because of the all up-regulate, we conjectured that the red mutation maybe more disease-resistant than ‘Zaosu’ pear.

### Cytoskeleton-related proteins

2 cytoskeleton-related proteins were detected as followed: D15 Os03g0718100 and D22 actin. D15 originates from leaf and D22 originates from fruit. D15 and D22 are all only exist in red bud mutation. Actin is the monomeric subunit of two types of filaments, and it is a globular roughly moonlighting protein in all eukaryotic cells. Actin participates in many important cellular processes of eukaryotic cells according to change the physical and chemical state of cells. The change of actin is a very import factor of cell division in the process of cellular division. Baluska et al. [30] found that cell wall pectin’s of meristematic maize root cells undergo rapid endocytosis in an F-actin-dependent manner. Starr and Han [31] reported that the actin cytoskeleton positioned nuclei in a variety of systems from yeast to plants and animal. In this study, D15 is one kind of actin which is named as Actin-1. It is a highly conserved protein to participate in many types of cell movement. It suggests that there may be a change in cytoskeleton and morphological of the red skin bud mutation. As actins in leaves and fruits are not the same, indicating that actin in different organs has its own specific expression.

### Amino acid metabolism-related proteins

There were 2 amino acid metabolism-related proteins detected as followed: D23 S-adenosylmethionine synthase and E4 putative methionine synthase. Both originate from fruit. S-adenosylmethionine synthase is an enzyme is the key enzyme to catalyze the synthesis of S-adenosylmethionie (SAM) from methionine and ATP. SAM is precursors of biosynthetic of ethylene [32] and polyamine [33], and participates in the process of transfer methyl and aminopropyl to nucleonic acid, protein and fat. In addition, SAM can be used as antioxidants. In this study, E4 only exists in Zaosu pear, but D23 was up-regulated in the mutation. We speculated that methionine cycle may be different in the red skin bud mutation.

### Antioxidant-related proteins

Only D11, Putative pyridoxine biosynthesis protein isoform A, was detected. The previous study showed that pyridoxine biosynthesis protein mainly related with plasma membrane and membrane system. It is also a rate-limiting enzyme in the process of vitamin B6 synthesis [34]. Vitamin B6 is a necessary element for all organisms, and it is cofactor of enzyme as an antioxidant in many metabolic pathways [35,36]. Since putative pyridoxine biosynthesis protein is up-regulation in leaf, it can increase the synthesis of vitamin B6, thus enhancing the ability of antioxidant in the red skin bud mutation.

### Calcium-related proteins

There was one calcium-related proteins protein (D10: Calreticulin) detected. It comes from leaf. Calreticulin is a multifunctional protein that binds Ca^2+^ ions. Calreticulin is located in storage compartments associated with the endoplasmic reticulum. It can be molecular chaperones of new synthesized protein. It has the function of regulate calcium balance, assists protein folding and processing, such as the endoplasmic reticulum custody protein, Calcium storage, signal transduction, regulate gene expression and apoptosis. In this study, Calcium was up-regulated in the red skin bud mutation. Thus, it is speculated that the toughness of cells increased, the ability of regulate and signal transduction was enhanced in the red skin bud mutation, leading to enhancement of the ability of resistance and stress response of plant.

### Gene ontology (GO) annotation and protein classification

**Figure 6:**
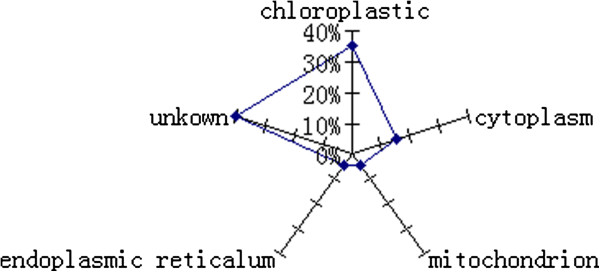
**The subcellular location of identified proteins.** Subcellular locations of the proteins were assigned according to the GO annotations and are expressed as percentages of the assigned proteins.

Subcellular locations of the proteins were assigned according to the GO annotations. (Figure 6) D4, D6, D7, D9, D13, D17 and E1 were according to chloroplast. Therein, D6, D7, D9 and E1 were annotated in chloroplast; D13 was annotated in chloroplast stromal; D17 was annotated in chloroplast thylakoid membrane. D15, D22 and D23 were classified in cytoplasm. D14 was annotated in mitochondrion and D10 was in end plastic reticulum lumen. Others were not annotated in Gene ontology.

### Chlorophyll content and the related enzyme activity

In order to reveal the different physiological traits between ‘Zaosu’ and its red bud mutation, we performed a series of physiological experiments. Chlorophyll II content, Rubisco content and PPO activity were measured. The result were showed in the Table 2.

**Table 2:** Chlorophyll content, rubisco content and PPO activity

	***Chlorophyll content*(*mg/g*)**	***Rubisco content*(*mg/g*)**	**PPO activity(*U·mg/ml*)**
‘Zaosu’ pear	33.4 ± 2.74a	72.71 ± 6.23a	16
red mutation	29.37 ± 3.21a	54.8 ± 5.33b	22

The data suggested that the chlorophyll content in ‘Zaosu’ pear was insignificantly higher, but do not have significant different with the red mutation while rubisco content in ‘Zaosu’ pear was significantly higher than that in the red mutation; PPO activity in the red mutation was significantly higher compared to ‘Zaosu’ pear.

## Conclusion

In summary, the optimized two-dimensional (2-D) gel electrophoresis system of pear leaf and fruit was set up, and applied to analyze the leaves and fruits protein. We have performed the first analysis of the proteomic changes in leaves and fruits of ‘Zaosu’ pear and its red skin bud mutation. We identified a series of proteins that are related mainly to the Photosynthetic and energy metabolism, stress resistance, diseases resistance, amino acid metabolism, cytoskeleton, and antioxidant. Our study provides important information on the use of proteomic methods for studying protein regulation of ‘Zaosu’ pear and its red skin bud mutation.

## Materials and methods

### Plant material

The leaves of ‘Zaosu’ pear and its red mutation were collected from the Germplasm Resources orchard of Northwest A and F University in mid April, 2009. The young fruits of ‘Zaosu’ pear and its red mutation were harvested from the experimental orchard of Weinan city, Shaanxi province in early May, 2009. The leaves and fruits frozen in liquid nitrogen immediately were stored at -80°C.

### Protein extraction

Protein extraction was performed according to Gallardo K [37]. Approximately 1.5-2 g of pear leaves or fruits mixed in PVPP, according to the proportion of 1/5, and 0.1 g DTT were ground to a fine powder in liquid nitrogen with mortar and pestle. Each microgram of protein powder precipitated with 15 μl of cold phenol extraction buffer [7 mol/l urea, 2 mol/l theorem, 4% (w/v) CHAPS, 65 mmol/l DTT, 0.2% (v/v) IPG buffer (pH 4-7; Bio-Rad, USA)]. After being vortex blended and incubated for 1 h at 4°C, proteins were collected by centrifugation at 12000 g for 40 min at 4°C and the supernatant stored at -20°C. Protein content was estimated by Bradford assay [38].

### Different sets of 2-DE gel electrophoresis

The IPG strips with pH 4-7 and pH 3-10 were tested, combined with bioelectric focusing (IEF) Procedure 1 (active rehydration at 50 V for 12 h at 17°C, S1 linear 250 V 30 min, S2 rapid 500 V 30 min, S3 rapid 1000 V 1 h, S4 linear 10,000 V 4 h, S5 rapid 10,000 V 60 kVh, S6 rapid 500 V 24 h) or bioelectric focusing (IEF) Procedure 2 (active rehydration at 50 V for 12 h at 17°C, S1 linear 250 V 30 min, S2 rapid 500 V 30 min, S3 rapid 1000 V 1 h, S4 linear 8,000 V 4 h, S5 rapid 8,000 V 60 kVh, S6 rapid 500 V 24 h). The results indicate that pH 4-7, 17 cm IPG strips combined with Procedure 1 was applied in the pear protein sample in this study.

### 2-DE gel electrophoresis and protein visualization

Protein (1000 μg, 350 μl) were loaded onto pH 4-7, 17 cm IPG strips with active rehydration for 12 h in [7 mol/l urea, 2 mol/l theorem, 4% (w/v) CHAPS, 65 mmol/l DTT, 0.2% (v/v) IPG buffer (pH 4-7), trace bromophenol blue]. Bioelectric focusing (IEF) procedure was performed at 25°C using the following setting: S1 linear 250 V 30 min, S2 rapid 500 V 30 min, S3 rapid 1000 V 1 h, S4 linear 10,000 V 4 h, S5 rapid 10,000 V 60 kVh, S6 rapid 500 V 24 h. After IEF, strips were equilibrated by gentle shaking for 15 min in equilibration buffer I [6 mol/l urea, 2% (w/v) SDS, 0.374 mol/l Tris–HCl pH 8.8, 20% (w/v) glycerol, 2% (w/v) DTT] and for an additional 15 min equilibrate in equilibration bufferII(DTT was replaced with 2.5% iodoacetamide for cysteine alkylation). The second-dimensional SDS-PAGE was performed with 12% polyacrylamide gel in Protein cell IEF (BIO-RAD, USA). The parameter of electrophoresis: 100 V 30 min, 180 V 6 h.

The gels were stained in the base of colloidal Coomassie Brilliant Blue G-250 [39]. Then, the gels were first washed by Milli-Q water for 3 times (5 min each time) and fasten in fixative solution [40% (v/v) ethanol, 10% (v/v) acetic acid, 10% (v/v) carbinol] for 1 h. After washed by mili-Q water for 3 times again, the gel stained by colloidal Coomassie Brilliant Blue [0.1% (w/v) CBB G-250, 10% (w/v) ammonium sulfate, 1.2% (v/v) phosphoric acid, 20% (v/v) ethanol] over night. The stained gels distained by distaining solution [10% (v/v) ethanol, 10% (v/v) acetic acid] until background clear so far.

### Image acquisition and cluster analysis

The gel images were acquired using an Powerlook2100XL optical density scanner and import into the PDQuest 8.0.1 (Bio-Rad, Hercules CA, USA) image software for analysis. A total of gels, resulting from three technical replicates for each biological replicate, were analyzed. The significance of changes of individual proteins between two physiological states was evaluated by the quantitative set with 1.5-fold change. Permut Matrix was used to conduct the cluster analysis for leaves and fruits, respectively, and the parameters were set as following: Dissimilarity: Pearson’s distance, Hierarchical: Ward’s Minimu Variance Meth, Used dataset: Normalize Rows (z-score).

### In-Gel tryptic digestion

After analysis by PDQuest image software, differential protein spots were excised from the preparative gels and stored in 2 ml eppendorf pipes. The gel pieces destained with 300 μl 100 mmol/l NH_4_HCO_3_ and 30% ACN (acrylonitrile). After removed the distaining buffer using 100% ACN, the gel pieces were lyophilized by lyophilizer. The dry gel pieces were rehydrated in 5 μl solution containing 2.5-10 ng/μl trypsin (Promega, Madison, WI, USA) for approximately 20 h. After taking hydrolysate out, they remained peptides was extracted in 100 μl of 60% CAN by sonication. Extracts were pooled together and lyophilized. The resulting lyophilized tryptic peptides were kept for mass spectrometric analysis.

### MALDI-TOF-TOF/MS Analysis

MS spectra analysis for peptides obtained using the 4800 Plus MALDI TOF/TOFTM Analyzer (Applied Biosystems, USA). Analysis completed on behalf of institute of Biochemistry and Cell Biology Shanghai Institute for Biological Sciences, Chinese Academy of Sciences.

### Database search and protein identification

The MS spectral data obtained using GPS Explore software for analysis, and the results of each sample integrate together into one file. The results were searched against the NCBInr database using the software MASCOT (Matrix Science, London, U.K.). The parameter settings of MASCOT were as the followings: trypsin as digesting enzyme with 1 missed cleavage allowed; search type set to peptide mass fingerprint; green plant set as search species; peptide mass tolerance set to 100 ppm; fragment tolerance set to ± 0.4 Da; carbamidomethyl C set as fixed modification; monoisotopic mass values set as protein quality; peptide charge state ion source set to +1; pI and MW is not required.

### Measurement of chlorophyll content

Chlorophyll content of pear leaves was determined according to Gao JF [40]. 0.1-0.2 g of pear leaves were powdered with 0.5 ml acetone. Then used 10-15 ml 80%(v/v) acetone washed the powder into centrifuge tube and digested over night. The extract diluted 10-fold and measured the absorbance of 665 nm and 649 nm. Used following formula calculate chlorophyll content

$$\begin{array}{c} \text{Chlorophyll content}\;\left(\text{mg}/g\right)\\ \;=\frac{\left(20.29\times A645+8.05\times A663\right)\times V}{W\times 1000}\times n \end{array}$$

(A_645_: absorbance in 645 nm, A_663_: absorbance in 663 nm, V: volume of acetone, W: weight of leaves, n: dilution).

### Measurement of rubisco

Rubisco content of pear leaves was determined according to Lilley RM [41]. Briefly, 5 g of pear leaves were frozen and powdered in liquid nitrogen, with 10 ml extraction ice-cold extraction buffer (50 mmol l^-1^ pH 7.5 Tries–HCl, 1 mmol l^-1^ EDTA, 10 mmol l^-1^ MgCl_2_, 12.5% (v/v) glycerol, 10 mmol l^-1^ β-mercaptoethanol, 1% PVP). The extract was stored at 4°C for 1 h, and then centrifuged at 5000 g for 15 min. The resulting supernatant was the crude enzyme extract. 100 ul crude enzyme extract added 1 ml brand ford working solution and placed in room temperature for 10 minutes. The content of Rubisco was spectrophotometrically monitored at 595 nm. The 100 ul PBS mixture 1 ml brand ford working solution was used as the blank [38].

### Assay of polyphone oxidase activity

The assay of PPO activity was conducted following the method by Kevin C et al. [42]. Fruit flesh tissues (10 g) were collected and homogenized with 25 ml of ice-cold extraction buffer (100 mmol l^-1^ sodium phosphate, pH 6.4), containing 0.5 g of polyvinyl polypyrrolidone (PVPP). The homogenate was centrifuged (4°C, 15,000 g, 50 min) and the supernatants were analyzed immediately. PPO activity was measured by incubating 0.5 ml of enzyme preparation in 3 ml of buffer substrate (100 mmol l^-1^ sodium phosphate (pH 6.4), and 500 mmol l^-1^ catechol) and monitoring the change of absorbance at 398 nm for 10 s. The specific activity was expressed as U mg l^-1^ protein, while the unit was defined as 0.001 of ΔOD_398_ min^-1^.

## Competing interests

The authors declare that they have no competing interests.

## Authors’ contributions

The first two authors contributed equality to this work. MH designed and carried out experiments analyzed data and wrote the manuscript. ZHQ performed the physiology experiments and participated in the writing of the manuscript. PZ participated in the MS analysis and assisted in the analysis of MS data. LFX conceived the study and participated in its design and assisted with the writing of manuscript. JKZ conceived of, designed and coordinated the study. All authors read and approved the final manuscript.
